# Lightweight Vehicle Detection Based on Improved YOLOv5s

**DOI:** 10.3390/s24041182

**Published:** 2024-02-11

**Authors:** Yuhai Wang, Shuobo Xu, Peng Wang, Kefeng Li, Ze Song, Quanfeng Zheng, Yanshun Li, Qiang He

**Affiliations:** School of Information and Electrical Engineering, Shandong Jiaotong University, Jinan 250357, China; 22208013@stu.sdjtu.edu.cn (Y.W.);

**Keywords:** artificial intelligence, deep learning, object detection, vehicle detection, lightweight

## Abstract

A vehicle detection algorithm is an indispensable component of intelligent traffic management and control systems, influencing the efficiency and functionality of the system. In this paper, we propose a lightweight improvement method for the YOLOv5 algorithm based on integrated perceptual attention, with few parameters and high detection accuracy. First, we propose a lightweight module IPA with a Transformer encoder based on integrated perceptual attention, which leads to a reduction in the number of parameters while capturing global dependencies for richer contextual information. Second, we propose a lightweight and efficient multiscale spatial channel reconstruction (MSCCR) module that does not increase parameter and computational complexity and facilitates representative feature learning. Finally, we incorporate the IPA module and the MSCCR module into the YOLOv5s backbone network to reduce model parameters and improve accuracy. The test results show that, compared with the original model, the model parameters decrease by about 9%, the average accuracy (mAP@50) increases by 3.1%, and the FLOPS does not increase.

## 1. Introduction

Although cars have brought us great convenience, problems such as traffic congestion and traffic accidents have become increasingly serious as the number of vehicles continues to increase. Intelligent traffic management and control systems can effectively solve the above problems [1,2,3]. Vehicle detection algorithms are an important part of intelligent traffic management and working systems. Therefore, vehicle detection algorithms have attracted much attention and become a popular research direction in the field of computer vision.

The traffic environment is complex and varied, including complex backgrounds, diverse vehicles, and changing traffic flows and road conditions. This complexity increases the difficulty of traffic target recognition, which needs to cope with variable scenarios, differences in vehicle shapes, and occlusion situations, as well as real-time availability. Therefore, a good vehicle detection algorithm is essential. At present, the YOLO series is one of the most excellent algorithms in single-stage detection; it has the characteristics of a smaller parameter count, high detection accuracy, and high speed. Therefore, it is widely used in vehicle inspection. Some researchers have chosen to use the original YOLO algorithm [4,5] for vehicle target detection tasks, which achieves a higher detection accuracy but also incurs high computational costs. Although the YOLOtiny model has relatively small parameters and computational complexity, it has a low detection accuracy and cannot detect vehicles effectively. In [6], the model’s ability to capture information about small objects was enhanced by introducing a new feature fusion layer and subsequent attention mechanism into YOLOv5, which improved the accuracy of the target detection. However, improving the model structure made it more complex and did not reduce the number of parameters. In [7], they incorporated the small object detection header into YOLOv5 and used a Transformer encoder block instead of some convolutional and CSP blocks to enhance the model’s ability to capture global and contextual information, which significantly improved the model’s performance but increased the model’s computational complexity (FLOPS) by 18.3% compared to the original model. There are also research algorithms [8,9] that utilise lightweight networks such as MobileNet or EfficientNet instead of YOLO’s entire backbone network. This approach significantly reduces the algorithmic parameters; however, their accuracy is significantly lower compared to the original algorithm.

Despite the importance of the above improved vehicle detection algorithm based on YOLOv5, there are still some problems. The Transformer encoder has multiple layers of self-attentive coding with powerful long-range dependency modelling capabilities. Incorporating it into YOLOv5 can significantly improve the algorithm’s performance. However, due to the stacking of the coding layers and the self-attentive computation, an increase in computational cost comes with embedding it into the model. The backbone network of YOLOv5, which is utilised instead of a lightweight network, is a very effective method for reducing the model parameters. However, lightweight networks are simple in structure and relatively weak in feature extraction. When confronted with some complex scenes, they may have difficulty capturing the detailed features in the scene.

In order to solve these problems, we propose an improved method based on integrated perceptual attention for YOLOv5. The main contributions of this paper are as follows:

- A lightweight module (IPA) with a Transformer encoder is proposed, which uses a parallel two-branch structure, where one branch uses efficient attention to capture global information, while the other branch uses convolutional attention to capture local information. The two-branch structure allows the model to better capture global and local information and obtain richer contextual information.
- A lightweight and efficient multiscale spatial channel reconstruction (MSCCR) module is proposed, which consists of only one efficient convolution and one ordinary attention, without excessive computational redundancy and parametric quantities. And an efficient module was designed by combining the multiscale spatial channel reconstruction (MSCCR) module with the C3 structure in YOLOv5. It is named the C3_MR module.
- The integration of the IPA module and C3_MR module into the YOLOv5s backbone network. Compared to the original model, the mAP@50 increases by 3.1% and the parameters decrease by about 9% with no increase in FLOPS.

## 2. Related Work

### 2.1. Vehicle Detection

Vehicle detection methods can usually be classified into two main categories: traditional methods and deep learning-based methods.

In traditional vehicle detection methods, manually designed features such as Scale Invariant Feature Transform (SIFT), Haar features [10], and the Histogram of Oriented Gradients (HOG) [11] are usually utilised to identify vehicles. These methods rely on manually designed rules to extract features and then use shallow machine learning models to perform vehicle detection tasks [12]. In [13], a new technique for vehicle detection in traffic scenes was proposed, which is based on frame differencing and a colour analysis of the foreground region (moving region). By using different colour channels and segmenting them according to the vehicle colour threshold in the image background, cars can be effectively distinguished from their surroundings. In [14], a symmetry-based vehicle detection method was proposed that exploits the symmetry feature of the vertical centreline at the rear of the vehicle. They achieve this by finding regions of interest (ROIs) that contain vehicles, in images with a high level of symmetry. In [15], a vision system for brake light detection during the daytime, using a travelling video recorder, was proposed. The operation of the system can be divided into two key steps. First, it uses symmetry verification of the tail lights to determine the presence of a vehicle in front of it. Then, once the position of the vehicle is confirmed, it combines luminance and radial symmetry features to detect the state of the brake lights. In [16], an algorithm was proposed to address an innovative technical challenge in visual analytics. The technique employs Hue, Saturation, and Lightness (HSV) colour segmentation combined with support vector machines (SVM) in order to detect moving emergency vehicles in traffic surveillance cameras. However, the above methods usually require the manual selection of features and the design and training of classifiers based on specific detection targets. The manual selection of features and classifier design require a high level of manual processing. Moreover, the maximum processing speed of vehicle detection by traditional methods does not exceed 40 FPS [17]. Therefore, it is not suitable for roads with fast travelling speeds.

With the continuous development of deep learning, traditional methods are gradually being replaced by deep learning-based target detection methods. These methods are divided into two main types. One is two-stage object detection algorithms such as R-CNN [18], Fast R-CNN [19], Faster R-CNN [20], and Mask R-CNN [21]. Suggested regions are first selected using a selective search algorithm and then categorised and regressed in the suggested regions. The other is one-stage target detection algorithms, such as SSD [22] and YOLO [23], which reduce the target detection task to a regression problem by eliminating the generation of suggestion regions and directly predicting the classes and locations of different objects. A two-stage detection algorithm is more accurate compared to a one-stage algorithm, but the model structure is more complex and the model parameters are much higher than the one-stage algorithm. Therefore, vehicle detection algorithms are generally dominated by single-stage target detection algorithms.

### 2.2. YOLOv5

The YOLO (You-Only-Look-Once) algorithm is the first single-stage detector capable of processing an image in one go and directly predicting the bounding boxes and categories of the objects in the figure. The YOLOv1 is fast but not very effective for objects that are close together and on small targets. The YOLOv2 algorithm adds an anchor mechanism, uses multiscale training, a fusion of shallow features and deep features, adds fine-grained features, and improves the problem of the poor detection of small targets. YOLOv3 further improves its performance by introducing a deeper feature extraction network (Darknet-53) to extract higher-level features [24]. YOLOv4 uses CSPDarknet53 as its backbone network and introduces a series of optimisations and innovations that reduce the number of parameters and FLOPS values of the model, ensuring accuracy while reducing the model’s size [25,26]. YOLOv5 has been optimised from YOLOv4; it uses a smaller model size and maintains excellent performance [27,28].

**Figure 1:**
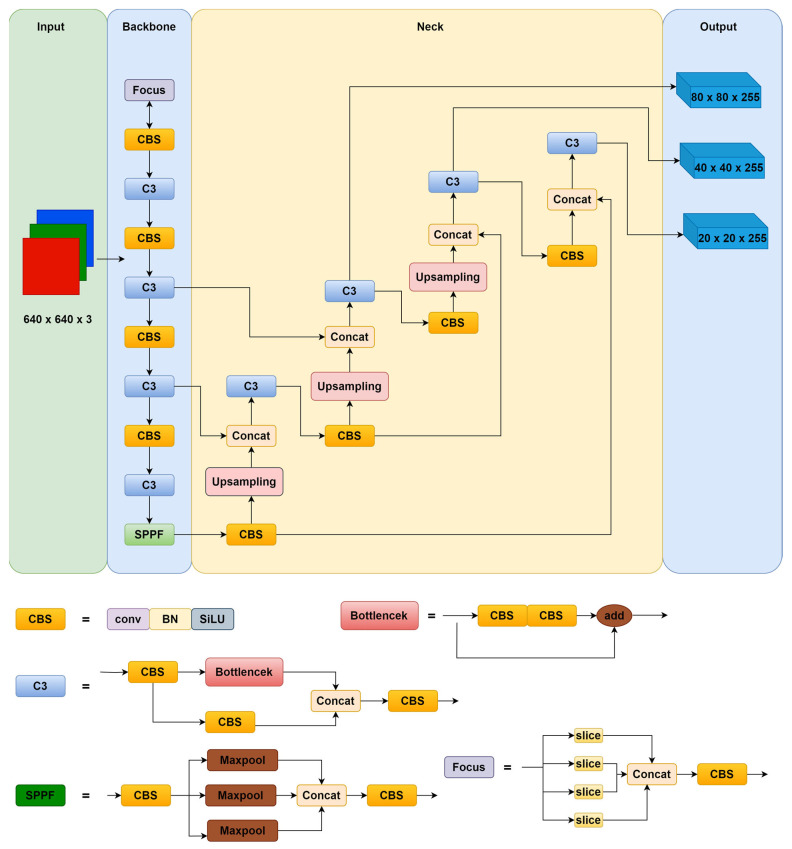
YOLOv5s network structure.

Depending on the depth of the model and the width of the feature map, YOLOv5 can be divided into four models: the YOLOv5s, the YOLOv5m, the YOLOv5l, and the YOLOv5x. These model sizes are shown in Table 1. From the table, it can be seen that the number of YOLOv5s parameters and the FLOPS are the smallest in these models. In this paper, we propose an approach aimed at achieving a lightweight design for the model, i.e., minimising the number of model parameters without increasing the computational complexity of the model. Therefore, the YOLOv5s is chosen as the benchmark model in this paper. The YOLOv5s network structure is shown in Figure 1, and the YOLOv5 family of models uses a consistent network structure. They consist of the following components: input, backbone, neck, and output.

The inputs include mosaic data enhancement, image size processing, and adaptive anchor frame calculation. The mosaic data enhancement enriches the background of a dataset by randomly selecting four images and randomly stitching, stacking, and scaling them. Image size processing adaptively adds a minimum black border to the images, resizing them to the same standard size. The adaptive anchor frame calculation outputs the bounding box based on the initial anchor frame, and then compares it with the real frame, calculates the gap, and then updates it in reverse, continually iterating the parameters to adaptively calculate the most suitable anchor frame value.

The backbone network consists of the Focus module, the CBS module, the C3 module, and the spatial pyramid pooling layer (SPFF) module. The Focus module divides the input data into four chunks, each of which corresponds to two downsamples. These four pieces of data are merged together in the channel dimension and then subjected to a convolution operation to produce a downsampled feature map without a loss of information. The CBS module consists of three parts: a convolutional layer, a batch normalisation layer, and an activation function, Silu. The C3 module consists of several bottleneck residual structure modules and three CBS modules. The bottleneck residual structure module processes the input through two convolutional layers and then performs an additive operation with the original input while passing residual features without increasing the depth of the output. The SPPF module is a spatial pyramid pooling layer [29], which is proposed on the basis of the SPP module to expand the sensory field, fuse local and global features, and enrich feature information [30].

The neck uses a combination of the Feature Pyramid Network (FPN) and Path Aggregation Network (PAN) [31], which help to enhance feature fusion. The FPN is responsible for passing top feature maps with strong semantic features to bottom feature maps, while PAN passes strong localisation features from bottom feature maps to top feature maps.

The output consists of three detection layers, using the Generalized Intersection over Union (GIOU) as the loss function of the bounding box and NonMaximum Suppression (NMS) [32], which employs a multiscale detection approach to detect objects of different sizes. Each output layer outputs corresponding vectors containing object category probabilities, object scores, and bounding box locations, and finally obtains the predicted borders and categories of the target objects and labels them on the original image.

### 2.3. Attention Mechanism

The method of focusing attention on the critical areas of an image and ignoring the unimportant parts is known as the attentional mechanism. The attentional mechanism is similar to that of the human visual system, through which the object detection model is able to determine which objects in the image are important and obtain their locations [33]. The attentional mechanism can be viewed as an adaptive weighting process that adjusts the feature weights according to the importance of the inputs. Attentional mechanisms have played an important role in image classification [34,35], object detection [36,37], face recognition [38,39], semantic segmentation [40,41], and other fields.

Some classical attentions include SE attention, CBAM attention, and CA attention [42]. The SE attention module is the first channel attention proposed by Momenta, which has at its core a squeeze module and an excitation module. Global information is collected through the squeeze module, channel relationships are captured using the excitation module, and the importance of each channel is learnt to adjust the channels of the input features. The CABM attention is an attention module proposed by Woo et al. It consists of two separate sub-modules, the Channel Attention Module (CAM) and the Spatial Attention Module (SAM). The CAM helps to establish the interdependencies between feature channels, whereas the SAM focuses on the areas of the feature map that are most relevant to the classification task. CA attention creates two one-dimensional average poolings for encoding global information in two spatial dimensions and capturing long-range interactions in different dimensions. Such a design not only preserves precise location information, but also exploits long-range dependencies by encoding inter-channel and spatial information.

Since the introduction of self-attention into computer vision, it has grown rapidly in the field with great success [43]. A novel type of cross-attention, i.e., row attention and column attention, is used in CCNet [44] to collect the contextual information of all pixels on that pixel cross-path and, through further cyclic operations, the global dependencies are made available to each pixel point.

DETR employs a Transformer architecture that applies self-attention to the task of target detection by mapping the objects in an image directly to target frames and categories for prediction, without the need for traditional anchor frames or suggestion regions, in order to achieve end-to-end target detection. The Swin Transformer [45] uses a sliding-window based attention that restricts attention computation to a single window while still allowing the model to learn information across windows, saving computation power and enabling its access to global and local information.

## 3. Materials and Methods

### 3.1. YOLOV5s Improvements

**Figure 2:**
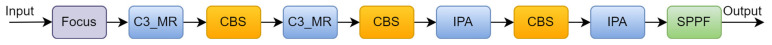
Improved backbone network.

We redesigned the backbone network of YOLOv5s using integrated perceptual attention and a C3_MR structure. The design principles follow the ideas of MobileViT [46], with a combination of convolution and self-attention. Specifically, the C3_MR structure is first used to aggregate shallow features instead of the two C3 structures in the backbone network. The integrated perceptual attention is then used instead of the last two C3 structures to aggregate deep features. This not only reduces the parameters of the model, but also achieves hierarchical feature learning, gradually extracting different levels of information from shallow to deep, making the model more expressive. The improved backbone network is shown in Figure 2.

### 3.2. Integrated Perception Attention Module (IPA)

**Figure 3:**
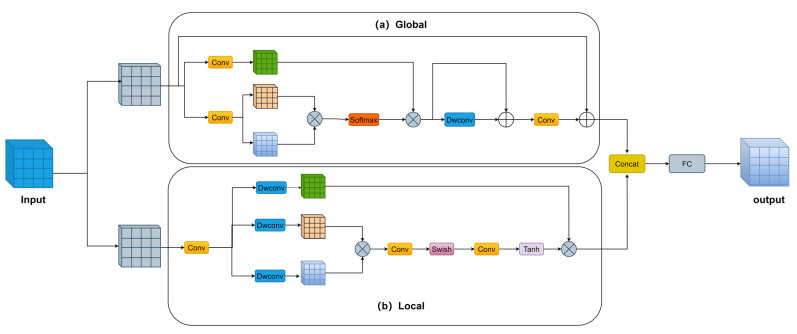
Integrated perception attention structure (IPA).

The Transformer encoder mainly consists of multiple layers of self-attentive coding, and its computation also originates from multiple coding layers. The Transformer encoder enhances the model’s ability to capture global information, while at the same time incurring high computational cost. To address this problem, we propose the integrated perceptual attention module (IPA), which employs a parallel two-branch structure, where one branch uses efficient attention to capture global information while the other branch uses convolutional attention to capture local information. The attention used in both branches is lightweight attention, which does not result in a huge increase in the model’s parameters and computational complexity. In addition to this, we have incorporated the idea of grouping into the integrated perceptual attention module. The structure of the integrated perceptual attention module is shown in Figure 3.

Global branching: Global branches use a simple and efficient Inverse Residual Moving Block (IRMB) [47] to aggregate global information, and the IRMB structure is shown in Figure 3a. This module is similar to the inverse inverted residual structure (IRB) in lightweight CNNs. IRB uses an extension layer to map feature dimensions to a higher dimensional space and uses depth-separable convolution to obtain more information in that higher dimensional space. IRMB replaces the depth separability in IRB with an efficient operator. The efficient operator consists of improved window attention and depth-separable convolution. Such a design not only incorporates CNN-like local feature modelling efficiency and Transformer-like dynamic modelling capabilities to learn long-distance interactions, but also does not impose unaffordable costs on the model.

Local branch: The local branch uses an attention-style convolution operator (AttnConv) [48] to aggregate local information, and the AttnConv structure is shown in Figure 3b. AttnConv, like the standard attention operation, first obtains Q, K, and V using linear transformations. Then, context-aware weights are generated by a nonlinear operator. The difference is that AttnConv obtains Q, K, and V and then aggregates the local information of Q, K, and V using depth-separable convolution, respectively, and the depth-separable convolution weights are shared globally. AttnConv uses Swish too, in addition to introducing Tanh. The use of dual nonlinear operators yields higher-quality context-aware weights to enhance local features.

Specifically, in integrated perceptual attention, we first introduce the idea of grouping into integrated attention, similar to group convolution [49,50], by dividing the input channels into n groups, which reduces the number of parameters and the computational complexity. Then, the grouped features are fed into the global branch and local branch for information aggregation, respectively. Finally, the outputs of the global and local branches are fused. Specifically, the output of the global branch and the output of the local branch are spliced in the channel dimension. The fully connected layer will then be utilised to increase the interaction between the different channels and reduce the context loss due to grouping.

### 3.3. MultiScale Spatial Channel Reconstruction Module (MSCCR)

**Figure 4:**
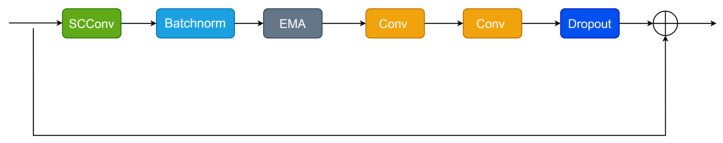
Multiscale spatial channel reconstruction (MSCCR).

**Figure 5:**
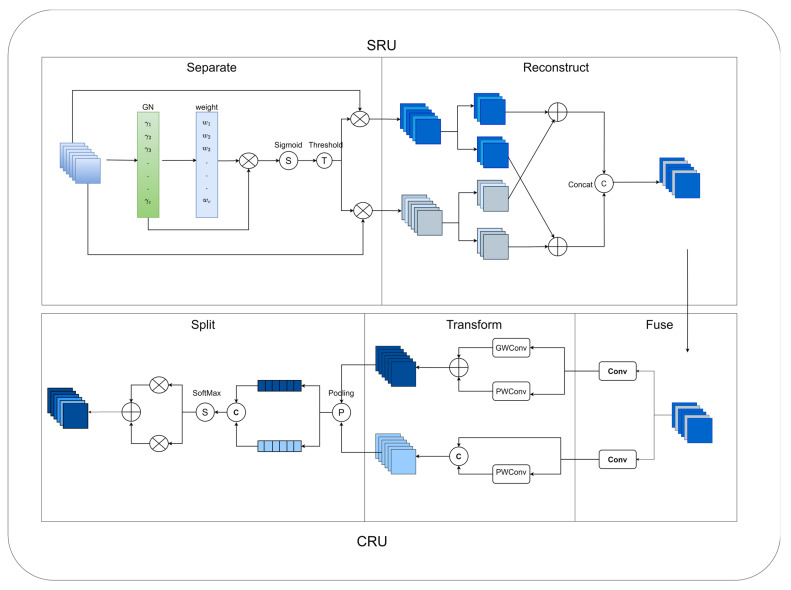
Spatial and Channel reconstruction Convolution (SCConv).

The structure of the multiscale spatial channel reconstruction module is shown in Figure 4. The multiscale spatial channel reconstruction module is centred on Spatial and Channel reconstruction Convolution (SCConv) [51]. Spatial and Channel reconstruction Convolution (SCConv) is an efficient convolution module that can effectively reduce computational redundancy and facilitate the learning of representative features. As shown in Figure 5, it consists of an SRU (Spatial Reconstruction Unit) and a CRU (Channel Reconstruction Unit). The SRU (Spatial Reconstruction Unit) uses separation and reconstruction to separate information-rich feature maps from information-poor feature maps and reconstruct the feature map weights. The CRU (Channel Reconstruction Unit) uses separation, transformation, and fusion strategies to extract rich feature information using inexpensive operations. The parameters of the standard convolution are calculated as

(1) $$P_{c}=C_{in}\times C_{out}\times k\times k$$

where $C_{in}$ and $C_{out}$ are the number of input and output channels and k is the convolution kernel size.

The parameters of the SCConv (Spatial and Channel reconstruction Convolution) are calculated as follows:

(2) $${\;P}_{sc}=1\times 1\times \alpha C_{in}\times \frac{\alpha C_{in}}{\gamma }+k\times k\times \frac{\alpha C_{in}}{g\gamma }\times \frac{C_{out}}{g}\times g+1\times 1\times \frac{\alpha C_{in}}{\gamma }\times C_{out}\;+\left(1-\alpha \right)C_{in}\times \frac{\left(1-\alpha \right)C_{in}}{\gamma }+1\times 1\times \frac{\left(1-\alpha \right)C_{in}}{\gamma }\times \left(C_{out}-\frac{1-\alpha }{\gamma }C_{in}\right)$$

where α, γ, and g are the hyperparameters of SCConv and k is the size of the convolution kernel, typically α = 1/2, γ = 2, and g = 2.

Standard convolution parameters are compared to SCConv parameters with an equal number of input channels, output channels, and the same convolution kernel size:

(3) $$\frac{P_{c}}{P_{sc}}\approx 5$$

The SCConv (Spatial and Channel reconstruction Convolution) parameter is a fifth of the standard convolution. So, using the SCConv as the core of the multiscale spatial channel reconstruction module can effectively reduce the number of parameters.

**Figure 6:**
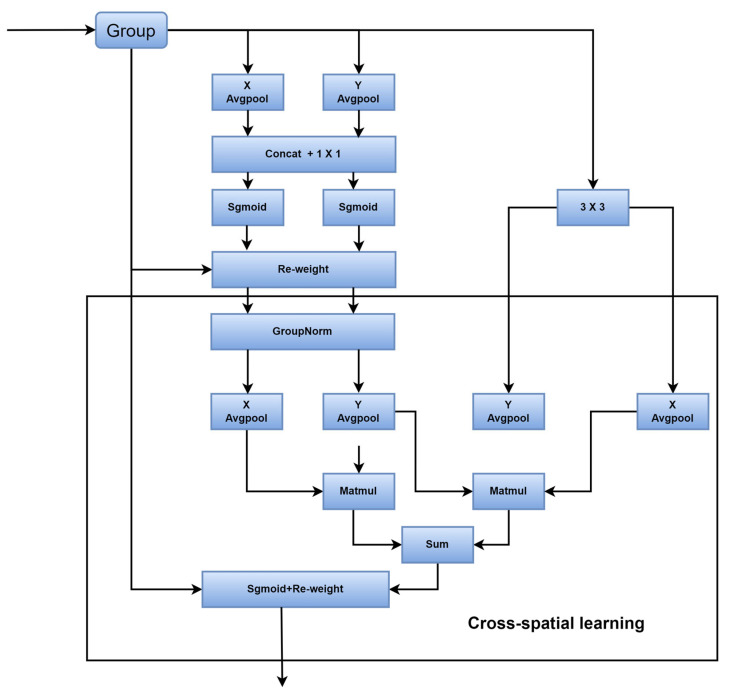
Efficient multiscale attention (EWA).

In addition to this, we add a type of efficient multiscale attention (EMA) [52] to the multiscale spatial channel reconstruction module. The EMA has a parallel three-branch structure, as shown in Figure 6. Different branches use different scales of convolution in order to facilitate the acquisition of multiscale spatial information and the establishment of long- and short-term dependencies. A richer set of features is then aggregated through the method of cross-spatial information aggregation. It is a lightweight attention; introducing it into the module does not add parameters and compensates for the fact that the convolution is strong locally but not globally.

### 3.4. C3_MR Build

The YOLOv5 backbone network is dominated by the C3 structure. The core of the C3 structure is a bottleneck residual structure module. The bottleneck residual structure module reduces the parameters of the backbone network by using the method of mapping features into a low-dimensional space. This reduces the number of parameters but also results in a loss of feature information. Specifically, we utilise the multiscale spatial channel reconstruction module proposed in Section 3.2 to replace the bottleneck residual structure module in the C3 structure, and call it the C3_MR structure. The bottleneck residual structure module parameters are calculated as

(4) $$P_{B}=1\times 1\times C_{in}\times C_{in}\times e+k\times k\times C_{in}\times e\times C_{out}$$

where $C_{in}$ and $C_{out}$ are the number of input and output channels, k is the convolution kernel size, and e is the scaling factor, usually 0.5.

The parameters of the multiscale spatial channel reconstruction module are calculated as

(5) $$P_{MR}=P_{sc}+1\times 1\times C_{in}\times C_{in}\times e+k\times k\times C_{in}\times e\times C_{out}$$

where $P_{sc}$ is the parameter of the SCConv, $C_{in}$ and $C_{out}$ are the number of input and output channels, and k is the convolution kernel size. e is the scaling factor, which is usually 0.5.

It is assumed that the bottleneck residual structure module and the multiscale spatial channel reconstruction module have the same number of input and output channels and that their convolution kernel size 3 × 3. The bottleneck residual structure module’s parameters can be compared to the multiscale spatial channel reconstruction module’s parameters:

(6) $$\frac{P_{B}}{P_{MR}}\approx 1.8$$

**Figure 7:**
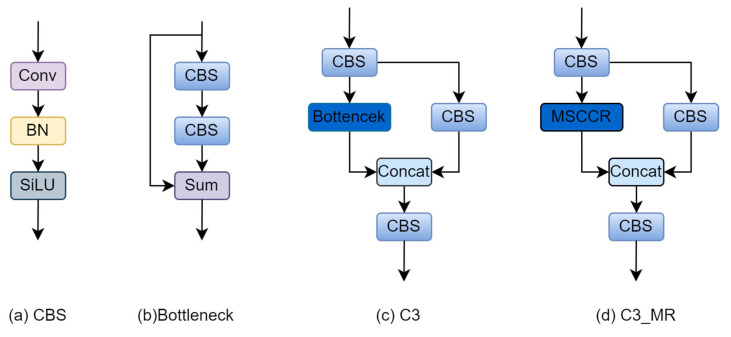
C3_MR.

The multiscale spatial channel reconstruction module is 1.8 times smaller than the bottleneck residual structure module. The parameters of the multiscale spatial channel reconstruction module in relation to the bottleneck residual structure module are shown in Table 2. The structure of C3_MR is shown in Figure 7.

## 4. Results

### 4.1. Dataset

This paper uses the UA-DETRAC dataset [53,54,55], a large open source resource for vehicle detection and tracking. The UA-DETRAC dataset is composed of road video surveillance from 24 different locations, covering a wide range of weather conditions, including sunny, rainy, and cloudy weather, and nighttime conditions. The types of vehicles in the dataset were divided into four categories, including cars, buses, lorries, and other types of vehicles. A total of 8250 vehicles and 1.21 million target objects were labelled.

We performed a frame extraction operation on the UA-DETRAC dataset, extracting the original video every 10 frames. Doing so, not only reduces the sample size of the dataset and reduces the training time of the model, but also prevents data redundancy. We selected 8639 images from the original training set to create a new training set, and chose 2231 images from the original validation set to create a new dataset.

### 4.2. Experimental Equipment and Evaluation Indicators

This experiment employs the Ubuntu 20.04 LTS operating system with an Intel Xeon Gold 6330 CPU, 128 GB of RAM, and an RTX 3090 GPU with 24 GB of VRAM. The deep learning framework used is PyTorch 1.10.1 with CUDA 11.8. The batch size for each training batch is 32, and a total of 100 training epochs are conducted.

To assess the performance improvement of YOLOv5s, the primary evaluation metrics used are the mean average precision (mAP@0.5) and the model’s parameter count.

The formula for calculating the average accuracy of *n* classes is as follows:

(7) $$mAP@50=\frac{1}{n}\sum_{i=1}^{n}\int_{0}^{1}P\left(R\right)dR$$

In the above equation, P and R represent precision and recall, respectively.

(8) $$P=\frac{TP}{TP+FP}$$

(9) $$R=\frac{TP}{TP+FN}$$

in which TP represents the number of true positives (correctly detected positive samples), FP represents the number of false positives (incorrectly detected negative samples), and FN represents the number of false negatives (incorrectly detected positive samples).

### 4.3. Comparisons

To validate the improved algorithm’s detection accuracy, we compared the enhanced YOLOv5s with the Faster-RCNN and SSD on the same dataset. The Faster-RCNN utilizes ResNet50 as its backbone network, while SSD uses VGG16 as its backbone network. As shown in Table 3, the parameter count of our improved algorithm is 15.5% of that of Faster-RCNN, with an accuracy 5.7% higher than Faster-RCNN. SSD has a parameter count 3.8 times that of our improved algorithm but has an accuracy 3.3% lower. Therefore, through a comparison with the one-stage detection algorithm SSD and the two-stage detection algorithm Faster-RCNN, our improved algorithm demonstrates a more accurate detection of vehicle targets.

To validate the performance of the improved backbone network, we compared it with some popular backbone networks such as MobileNetV2, MobileNetV3, and EfficientNet. We conducted experiments by replacing the backbone network of YOLOv5s with the aforementioned backbone networks. The experimental results are shown in Table 4. The improved backbone network achieved a mAP@50 that is 5.6% higher than MobileNetV2, 6.7% higher than MobileNetV3, and 4.1% higher than EfficientNet. The mAP@50:95 is also superior to mainstream backbone networks, and the parameter count is roughly the same. Through this comparison, it can be concluded that under nearly identical parameter counts, our improved backbone network outperforms popular backbone networks in terms of performance.

We also compared the improved algorithm with similar algorithms: YOLOv3-tiny, YOLOv4-tiny, and YOLOv5s (the original model). As seen in Table 5, YOLOv3-tiny has 1.4 times the parameter count of the improved model but a 6.7% lower mAP@50. The improved model reduces the parameter count by 9% and increases the mAP@50 by 3.1%. It also performs well compared to YOLOv6s and YOLOv7-tiny. This indicates that the improved algorithm also performs excellently against similar algorithms.

### 4.4. Comparison of Test Results

**Figure 8:**
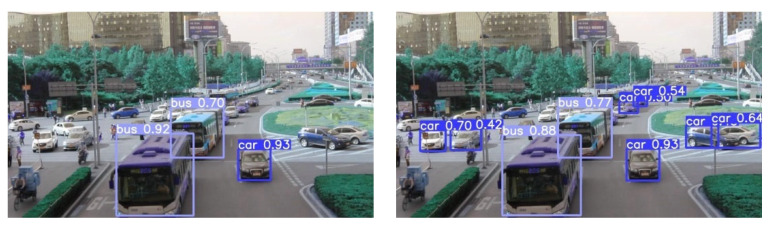

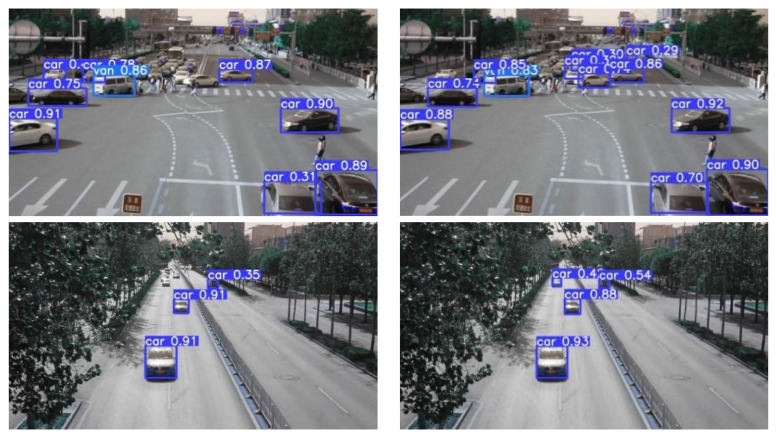
Detection results of YOLOv5s and improved model.

The visual results of vehicle detection in different scenarios for our improved model and YOLOv5 are shown in Figure 8. The left image represents the detection results of YOLOv5s, while the right image displays the detection results of our improved model. From Figure 8, it can be observed that the first and second images depict complex environments with a higher number of vehicles, while the third image represents a simpler environment with fewer vehicles. In both simple and complex environments, the number of vehicles detected in the left panel is less than in the right panel, and the detection accuracy is also generally lower than in the right panel. It can be concluded that the YOLOv5s algorithm has weak adaptability and a poor feature extraction ability in complex scenes, and is prone to low detection accuracy and missed detection problems. Our proposed method can improve the problems of YOLOv5s and better detect vehicles.

### 4.5. Grad-CAM Visualisation

**Figure 9:**
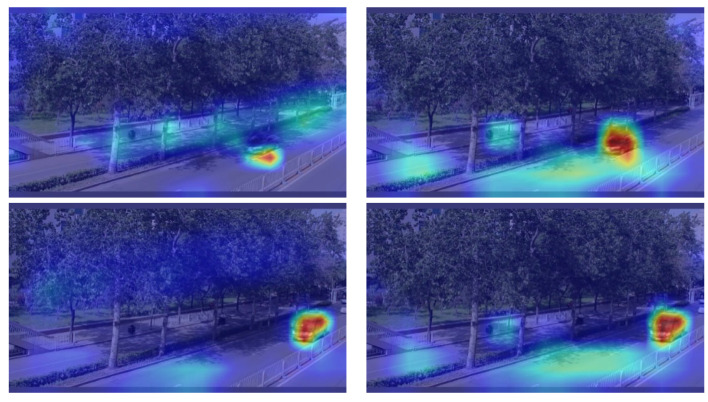
Grad-CAM visualisation results for YOLOv5s and improved model.

To further demonstrate the effectiveness of our method, we conducted Grad-CAM visualizations for YOLOv5s and the improved model, as shown in Figure 9. The left image in each pair represents the Grad-CAM visualization results for YOLOv5s, while the right image represents the Grad-CAM visualization results for the improved model. In the first image, it can be clearly observed that the improved model focuses more accurately on the area in which the object is located than YOLOv5s. In the second image, although both the improved model and YOLOv5s are able to locate the object’s region, the Grad-CAM map of the improved model is darker, which indicates that the improved model pays more attention to the object’s region, emphasising the importance of this region. Therefore, our proposed method can further fine-grain the features, making the model more accurate at locating and recognising objects.

### 4.6. Ablation Experiment

To demonstrate the impact of the improvements proposed, we conducted ablation experiments, as shown in Table 6. From the table, it can be observed that although C3_MR uses SCConv, a similar 3 × 3 convolution, it does not increase the model’s parameters and it improves detection accuracy. IAT effectively reduces model parameters and enhances model accuracy. When all these improvements are combined with YOLOv5s, they significantly enhance the algorithm’s accuracy while reducing the model’s parameter count.

## 5. Discussion

With the continuous development of automotive technology, vehicle detection algorithms are widely used in autonomous driving to capture images and video data around the vehicle using on-board cameras. Vehicle detection algorithms use this data to identify vehicle targets in images through complex computations and model analysis. Combined with on-board cameras and vehicle detection algorithms, the vehicle system is able to sense vehicle dynamics on the surrounding roads in real time. Moreover, vehicle detection algorithms combined with millimetre wave radar or LiDAR can improve the robustness and reliability of vehicle detection [56]. Some researchers have chosen to use the original YOLO algorithm in vehicle detection [4,5]. Although it can accurately detect vehicle targets, when applied to vehicle camera-like mobile terminal devices [57] it imposes a huge computational cost on the mobile terminal device and makes it difficult to maintain a high frame rate for real-time detection during runtime. YOLOtiny has smaller parameters and a smaller model size compared to the original YOLO algorithm, but it is less accurate and insufficient at detecting vehicle targets [30]. The method proposed in this paper can effectively reduce the model’s parameters and improve its accuracy. Therefore, the method can be used as a solution to the above problem. Due to the limitations of mobile terminal devices, we plan to conduct practical experiments in future studies to verify the performance of our proposed method in real applications. In addition, we will further optimise the algorithms to adapt them to the limitations of different hardware platforms and computing resources to ensure that efficient vehicle detection can be achieved on mobile terminal devices.

## 6. Conclusions

In order to address the issues of the complex structure and large hardware requirements of current vehicle detection algorithms, this paper proposes a lightweight vehicle detection model based on comprehensive perception attention. Two lightweight modules, the integrated perceptual attention module and the multiscale spatial channel reconstruction module, were designed and successfully integrated into the YOLOv5s algorithm.

The experimental results demonstrate that, compared to YOLOv5s, the improved algorithm achieved a 3.1% increase in its average precision on the UA-DETRAC dataset. In comparison to SSD, it outperforms it with a 3.3% higher mAP@50. When compared to Faster-RCNN, it also surpasses that algorithm with a 3.3% higher mAP@50. In comparison to other backbone networks, it achieves a 5.6% higher mAP@50 than MobileNetV2, 6.7% higher than MobileNetV3, and 4.1% higher than EfficientNet. Compared to similar algorithms like YOLOv3-tiny and YOLOv4-tiny, it achieves a 5.7% and 6.7% higher mAP@50, respectively. The improved algorithm exhibits excellent performance in various scenarios. This algorithm not only ensures higher accuracy but also effectively reduces computational costs, decreasing the demand for storage and computing resources. Therefore, it is well-suited for deployment in resource-constrained devices. Future focus can be on successfully deploying the improved model in resource-constrained embedded devices to achieve practical applications in the field of vehicle detection. This initiative will contribute to further enhancing the proposed algorithm and its methods to meet real-world requirements.

## Figures and Tables

**Table 1:** Comparison of different model sizes of YOLOv5.

Model	Size	Params (M)	FLOPs (G)
YOLOv5s	640 × 640	7.0	16.0
YOLOv5m	640 × 640	21.1	49.2
YOLOv5l	640 × 640	46.5	109.6
YOLOv5x	640 × 640	86.7	206.3

**Table 2:** Comparison of bottleneck and MSCCR parameter counts.

Model	Size	Params
Bottleneck	640 × 640	20,672
MSCCR	640 × 640	11,852

**Table 3:** Comparison of the different algorithms.

Model	Params (M)	FLOPS (G)	mAP@50
Faster-RCNN	41.3	60.5	0.508
SSD	24.1	30.5	0.532
our	6.4	15.6	0.565

**Table 4:** Comparison of the performance of different backbone networks.

Model	Backbone	Params (M)	FLOPS (G)	mAP@50	mAP@50:95
YOLOv5s	Mobileentv2	7.4	16.1	0.509	0.347
YOLOv5s	Mobileentv3	7.3	9.9	0.498	0.335
YOLOv5s	Efficientnet	7.0	13.4	0.524	0.347
YOLOv5s	Ours	6.4	15.6	0.565	0.384

**Table 5:** Performance comparison of different lightweight networks.

Model	Params (M)	FLOPS (G)	mAP@50	FPS
YOLOv3-tiny	8.7	12.9	0.498	-
YOLOv4-tiny	6.0	16.2	0.508	-
YOLOv5s	7.0	16.0	0.534	78.5
YOLOV6n	4.6	11.3	0.536	84.3
YOLOV7-tiny	6.0	13.0	0.472	98
Ours	6.4	15.6	0.565	52

**Table 6:** Results of ablation experiments.

Model	Params (M)	FLOPS (G)	mAP@50
YOLOv5s	7.0	16.0	0.534
YOLOv5s + C3_MR	7.0	15.4	0.557
YOLOv5s + IAT	6.4	16.2	0.547
YOLOv5s + C3_MR + IAT	6.4	15.6	0.565

## Data Availability

The original contributions presented in the study are included in the article, further inquiries can be directed to the corresponding author.
